# Proof-of-principle investigation of an algorithmic model of adenosine-mediated angiogenesis

**DOI:** 10.1186/1742-4682-8-7

**Published:** 2011-04-08

**Authors:** Francisco Azuaje, Frédérique Léonard, Magali Rolland-Turner, Yvan Devaux, Daniel R Wagner

**Affiliations:** 1 Laboratory of Cardiovascular Research, Centre de Recherche Public - Santé (CRP-Santé), L-1150, Luxembourg, Luxembourg; 2 Division of Cardiology, Centre Hospitalier, L-1210, Luxembourg, Luxembourg

## Abstract

**Background:**

We investigated an algorithmic approach to modelling angiogenesis controlled by vascular endothelial growth factor (VEGF), the anti-angiogenic soluble VEGF receptor 1 (sVEGFR-1) and adenosine (Ado). We explored its feasibility to test angiogenesis-relevant hypotheses. We illustrated its potential to investigate the role of Ado as an angiogenesis modulator by enhancing VEGF activity and antagonizing sVEGFR-1.

**Results:**

We implemented an algorithmic model of angiogenesis consisting of the dynamic interaction of endothelial cells, VEGF, sVEGFR-1 and Ado entities. The model is based on a logic rule-based methodology in which the local behaviour of the cells and molecules is encoded using *if-then *rules. The model shows how Ado may enhance angiogenesis through activating and inhibiting effects on VEGF and sVEGFR-1 respectively. Despite the relative simplicity of the model, it recapitulated basic features observed in *in vitro *models. However, observed disagreements between our models and *in vitro *data suggest possible knowledge gaps and may guide future experimental directions.

**Conclusions:**

The proposed model can support the exploration of hypotheses about the role of different molecular entities and experimental conditions in angiogenesis. Future expansions can also be applied to assist research planning in this and other biomedical domains.

## Background

Angiogenesis, the generation and development of new blood vessels from existing ones, is a fundamental complex process in health and disease [1,2]. The evolution of new blood vessel networks may be defined as the by-product of the division and migration of endothelial cells (ECs) in response to different physiological molecular conditions or pathological stress stimuli. Hypoxia, the deprivation of oxygen delivery to a tissue, is among such angiogenesis-triggering conditions. Hypoxia-induced angiogenesis is critical in the understanding of mechanisms underlying the evolution of tumours and cardiac damage. Angiogenesis requires the molecular signalling interplay between a plethora of growth factors, anti-angiogenic molecules and environmental stimuli [1]. Vascular endothelial growth factor (VEGF) is one of the most potent pro-angiogenic molecules activated in hypoxic conditions. VEGF binds to several receptors, such as the membrane-associated receptor VEGFR-1 or fms-like tyrosine kinase 1 (Flt1). A soluble form of VEGFR-1 (sVEGFR-1) traps circulating VEGF and prevents its binding to membrane receptors, thereby acting as a decoy receptor having anti-angiogenic properties [2,3]. This is a typical example of a molecule sharing dual roles in angiogenesis according to specific intra- and extra-cellular localization [4,5].

*In silico *models of angiogenesis have been investigated in unicellular and multi-cellular contexts chiefly through the implementation of numerical approaches, i.e. differential reaction equations [6,7]. Computational or algorithmic models define a second family of approaches. These are based on operational descriptions of molecular interactions and processes, e.g. sets of *if-then *rules, which are used to dynamically encode and execute the models [8,9]. Unlike traditional mathematical models, such as those based on reaction equations, algorithmic models can incorporate dynamic visualization capabilities at the individual cellular and tissue levels. Moreover, algorithmic models can integrate specific causal mechanistic information at the cell or multi-cell levels. Another key reason for selecting this methodology was that it does not require the precise approximation of mathematical parameters, such as concentration rates, which are required in traditional reaction models. This is particularly relevant to our problem due to the relative lack of quantitative information to allow us to implement more detailed models. Furthermore, at this stage we are mainly interested in assessing its potential as a simulation-based exploratory tool.

*In silico *models, in general, can recreate or mimic the initiation and development of blood vessel networks in different medically-relevant scenarios [10,11]. Mathematical and computational models have received relatively greater attention in the area of cancer research [12-15]. Within this area, several computational models based on cellular automata or agent-based systems have been proposed [13,15-19], which approximate diverse structural and functional aspects of cellular growth or angiogenesis. Furthermore, there is a need to implement models relevant to other biomedical settings, including those in which angiogenesis can play protective or therapeutic functions, e.g. myocardial infarction.

Our research group investigates the role of angiogenesis in the context of cardiac disease. In particular, we are interested in studying the regulation of angiogenesis to promote the treatment and repair of the ischemic heart. Apart from investigating the dynamic interaction between known pro- and anti-angiogenic factors, we aim to characterize the modulating effects of cardio-protective factors, such as adenosine (Ado). Previous research has shown how Ado can promote angiogenesis in ischemic tissue [16,20]. Moreover, Ado has been found to drive ECs proliferation, migration and subsequent vessel network development in the heart [21-23]. We and others have reported that Ado controls VEGF expression and activity [23-29]. We hypothesized that the effect of Ado on VEGF pathway may be a more complex phenomenon than simply an enhancement of expression. Therefore, to guide future *in vitro *experimental developments, we set out to investigate the roles that VEGF, sVEGFR-1 and Ado can play in angiogenesis using an algorithmic exploratory model.

We introduce here a computational model of sprouting angiogenesis in which the ECs divide and move to generate complex vascular networks through the integrated effect of VEGF and sVEGFR-1. We also tested the hypothesis that Ado promotes angiogenesis by simultaneously enhancing VEGF and reducing sVEGFR-1 activity. Our model mimics the generation of vascular networks under different experimental conditions, and enables the visualization of branching patterns and systems-based phenomena that approximate global *in vitro *and *in vivo *behaviours. The model was specified and implemented as a "rule-based" system, in which thousands of ECs and molecules autonomously and locally interact following fundamental mechanistic principles encoded as "if-then" rules. Such an interaction is performed in parallel to give rise to the observed emergent behaviours at the multi-cellular level. Our model can actually be defined as one belonging to the category of agent-based models (also known as individual-based models). Key features of this category are the heterogeneity of spatial states, the diversity of model components and their behaviours, and the application of decision-making rules at the local control level only.

We quantitatively assessed the effects and relationships between the model components, and generated testable predictions. These analyses were followed by *in vitro *experiments as a first step to estimate potential biological relevance and feasibility of the proposed computational model. In principle, the computational model enabled us to verify different hypotheses and led us to a deeper biological understanding. The results also provided insights that may suggest a possible reformulation of some aspects of our hypothesis about the combined effects of Ado, VEGF and sVEGFR-1.

## Methods

### Model foundations and study phases

A pivotal conceptual premise of our model is that the individual behaviour of the biological *entities *(ECs, VEGF, sVEGFR-1, Ado) may be synthesized by a set of algorithmic rules. Such rules specify the entities' local behaviour in response to the state of other entities and the environment. Thus, the rules encapsulate biological hypotheses about entity interactions (cell-cell, cell-molecule or cell-environment) in a computing format suitable to dynamic simulations. The rules are applied to each entity to update its state in a time- and space-specific fashion. Each state update may trigger the division and/or movement of an entity. At local and individual entity levels, such rule-driven transformation processing has little dynamic predictive value. However, the integration of individual entity behaviours in time and space leads to angiogenesis-like, branching patterns. This also means that in our models there is no specification or centralized control of global or collective behaviour. Our entities update their states only in response to their local environment (spatial *neighbourhood*, see Computational model specification).

In our models, a rule specifies the actions of an entity in response to its own (current) state or the state of its neighbourhood based on conditional programming statements. The application of such logic rules and the resulting transformation are conducted following a stochastic (non-deterministic) scheme. This stochastic behaviour is defined by a probability of actually executing the rule, which encodes the notion of random motility and response heterogeneity of biological entities [29,30]. The spatial environment is represented by a 2D grid (a matrix of *m *× *n *sites). Boolean variables are used to represent the presence or absence of an entity in each grid site. Such a grid can be interpreted as the digital, though rough, equivalent of a Matrigel assay, i.e. our angiogenesis *in vitro *assay. In our model, time is represented by simulation *cycles*. In each cycle, the system executes all the model rules and updates all entity states at each grid site. Each simulation (experiment) consists of a pre-specified number of cycles (see computational model specification).

**Figure 1:**
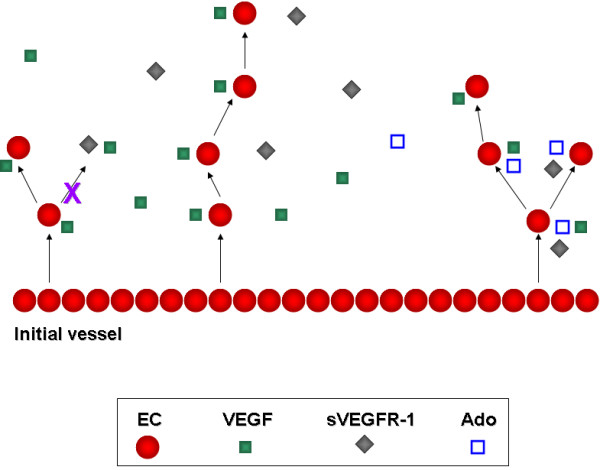
**Cartoon diagram of our angiogenesis model**. Model consisting of four entities: EC, VEGF, sVEGFR-1 and Ado. Sequence of events is visualised from the bottom to the top of the figure. Arrows are used to indicate EC division and migration to a new site.

Figure 1 is a cartoon diagram of our angiogenesis model consisting of four entities: EC, VEGF, sVEGFR-1 and Ado. This illustration is better interpreted from the bottom to the top. Arrows are used to indicate EC division and migration to a new site. At the beginning of a simulation, an initial set of ECs forms the "initial vessel", located at the bottom of the grid. The other entities are randomly distributed on the grid, which can be seen as the equivalent of an isotropic distribution of molecules at the start of an *in vitro *experiment. An EC "divides" and "moves" to a new site *If *a VEGF entity is present *And *a sVEGFR-1 is absent in the immediate EC's neighbourhood. Thus, a sVEGFR-1 will inhibit the birth of a new EC (symbolised in the figure with an arrow crossed with an X). Similarly, an EC can divide and move to a new site *If *Ado is present in the immediate EC's neighbourhood, independently of the presence of sVEGFR-1 (right side of figure). This defines a central hypothesis of our model: Ado promotes angiogenesis by enhancing VEGF activity and by antagonizing sVEGFR-1. This is because the presence of Ado would allow VEGF to exert its effect on EC independently of the presence of inhibitors. For additional information on design principles and computing implementation of rule-based or algorithmic models the reader may refer to [31,32].

**Figure 2:**
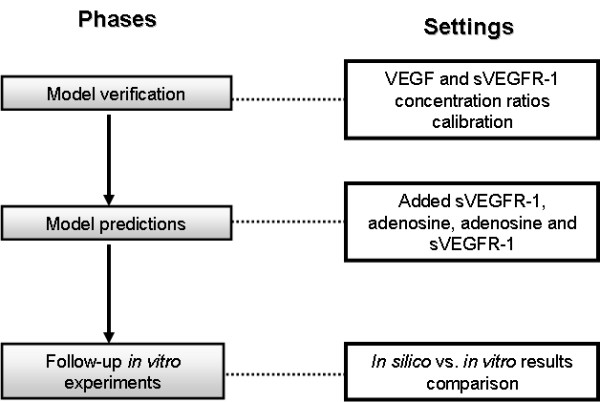
**Experimental and analytical phases of our investigation**.

Figure 2 summarizes the different phases of our investigation. In the first phase, model verification, we implemented a foundation angiogenesis model in which EC growth is controlled by VEGF and sVEGFR-1 only. Thousands of simulations allowed us to test different experimental conditions, i.e., different VEGF and sVEGFR-1 concentration values. This phase resulted in the definition of biologically plausible, calibrated models both in terms of the predicted outcomes (e.g., resulting angiogenic-like visual patterns) and quantitative parameter relationships. In this phase we identified the control model settings that were needed in subsequent research phases. In the prediction phase we tested the Ado-mediated angiogenesis hypothesis. Moreover, we conducted independent experiments in which the effects of adding sVEGFR-1, Ado and variable proportions of both entities were estimated. As an initial step to estimate the potential biological utility of our computational approach, we carried out *in vitro *experiments using culture media of preconditioned human primary macrophages treated (and untreated) with Ado on a MATRIGEL cultured human coronary artery endothelial cells (HCAEC) model. This was done in the presence (and absence) of additional exogenous sVEGFR-1. Although this phase does not actually represent an experimental validation of the model, it allowed us to compare the predicted global effects of added sVEGFR-1, Ado and combined Ado/sVEGFR-1 (relative to control conditions) with *in vitro *observations obtained at our laboratory.

### Computational model specification

**Figure 3:**
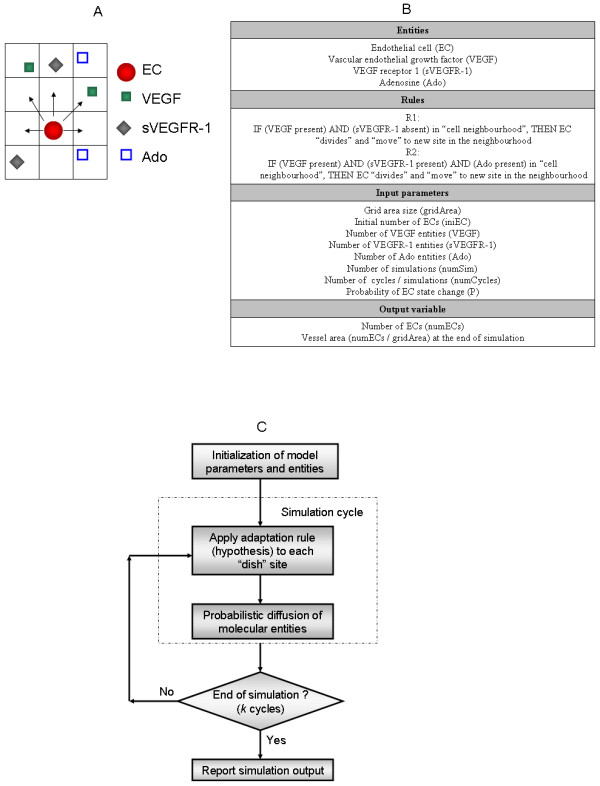
**Model specification**. A. Definition of the concept of EC neighbourhood. Arrows indicate the direction of possible moves of an EC at a particular step cycle. B. Definition of main model parameters and variables: Entities, model rules, input parameters, and output variables. Rules R1 and R2 were independently implemented in systems verification and prediction phases. C. Flow chart summarising model algorithm.

Figure 3 illustrates the main design components and processes of the models. The concept of EC neighbourhood is further illustrated with a cartoon representation involving the model entities. Arrows are used (Figure 3A) to show the directions in which an EC can move at a particular cycle step. The model is based on the interaction of 4 molecular entities: EC, VEGF, sVEGFR-1 and Ado. Two logical rules were independently implemented and investigated for different numerical parameters. The first rule (R1) in Figure 3B encodes the behaviour of EC, VEGF and sVEGFR-1 in control conditions only (Model Verification Results). The second rule (R2) defines the Ado-mediated model investigated. The input parameters of each simulation are: grid area size (gridArea, m × n sites on the grid), initial number of ECs (iniEC), number of VEGF entities (VEGF), number of sVEGFR-1 entities (sVEGFR-1), number of Ado entities (Ado), number of simulations (numSim), number of cycles per simulation (numCycles) and the probability of a EC moving to a new site after rule application (i.e., probability of effective rule execution). The iniEC parameter defines the length of the initial vessel located at the bottom of the grid (sprouting vessel). The output of each model simulation was assessed on the basis of the resulting vessel network area: numECs/gridArea, with numECs representing the total number of ECs observed at the end of a simulation. The output of sets of simulations was summarized with their mean values. Figure 3C describes the main steps implemented in a single simulation. After model parameters have been initialized, a simulation cycle starts by the application of the model rules to each site on the grid. After all entity states have been adapted, molecular entities on the grid are stochastically diffused, i.e., an entity moves to a (nearest) neighbour site randomly chosen. This diffusion process only applied to VEGF, sVEGFR-1 and Ado. These steps are repeated for numCycles.

### *In silico *experiments: implementation and execution

The models reported in this paper were implemented with the following input parameters: gridArea = 90E3 (300 × 300), iniEC = 300, numCycles = 300, numSim = 1000 and P = 0.05. Quantitative responses to different levels and relative proportions of VEGF, sVEGFR-1 and Ado were investigated as shown above. The reported simulations were implemented with 300 cycles/simulation. In ideal cell-growth conditions, this would be sufficient to allow the tip of a network to reach the top of the grid in a single simulation, i.e., maximum grid length. However, larger numbers of cycles reported very similar overall responses to those observed in models with numCycles = 300. This may be an indication of steady state response. To exemplify this point, Additional file 1 illustrates results from the Ado-treatment setting with numbers of cycles ranging from 300 to 1500.

### *In vitro *experiments

Cell culture: Peripheral blood mononuclear cells (PBMCs) from healthy volunteers (1 sample/person) were isolated by Ficoll gradient. Monocytes were purified by negative selection using the Monocyte Isolation Kit II (Myltenyi Biotec GmbH, Bergisch Gladbach, Germany) as described before [33]. Differentiation was achieved by adding 50 ng/mL M-CSF for 7 days. The obtained macrophages were then incubated with Ado and EHNA (10 μmol/L) (Sigma, Bornem, Belgium) to prevent Ado metabolism. LPS (from Escherichia coli 026:B6)) (Sigma) was used as cell activator. Conditioned medium was harvested and stored at -80°C until use.

*In vitro *angiogenesis assay: Human Coronary Artery Endothelial Cells (HCAEC, Lonza, Verviers, Belgium) were seeded on Growth Factor Reduced Matrigel™ (BD Bioscience, Erembodegem, Belgium) coated 48-well plates. Culture medium was made of a 1/1 mix of EBM2 medium (Lonza) containing 2% of Fetal Calf Serum and conditioned medium from macrophages treated with LPS and/or Ado as described above. In some cases, 10 ng/mL of sVEGFR-1 (R&D Systems, Oxon Abingdon, UK) was added in this culture medium 1 hour before the contact with HCAEC. After six hours, the formation of microtubules by HCAEC was blindly measured by three different investigators on three microscopic fields per culture well. This formation was evaluated by measurement of the vascular surface area using Aïda software (Kodak, Zaventem, Belgium).

### Ethics Statement

The sample acquisition protocol was approved by the local ethics committee (Comité National d'Éthique de la Recherché, CNER) and written informed consent was obtained from all volunteers.

### Statistics and software

Mean-based comparisons between independent groups were carried out with t-tests, and 95% confidence intervals were calculated around estimated means. Statistical analyses were done with Statistica v8 [34]. Model algorithm was implemented in the Java programming language.

## Results

### Model verification

**Figure 4:**
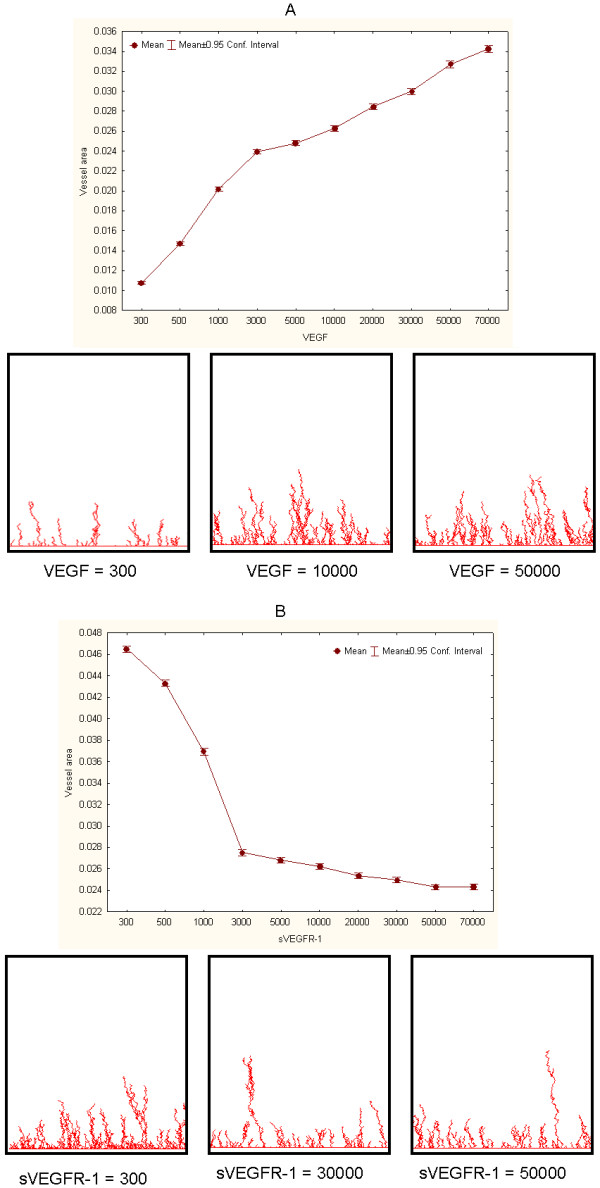
**Relationship between molecule concentration values and vessel network areas**. Illustrative examples of A. sVEGFR-1 = 10000 and VEGF variable. B. VEGF = 10000 and sVEGFR-1 variable. Panels show examples of snapshots of graphical outputs of simulations.

**Figure 5:**
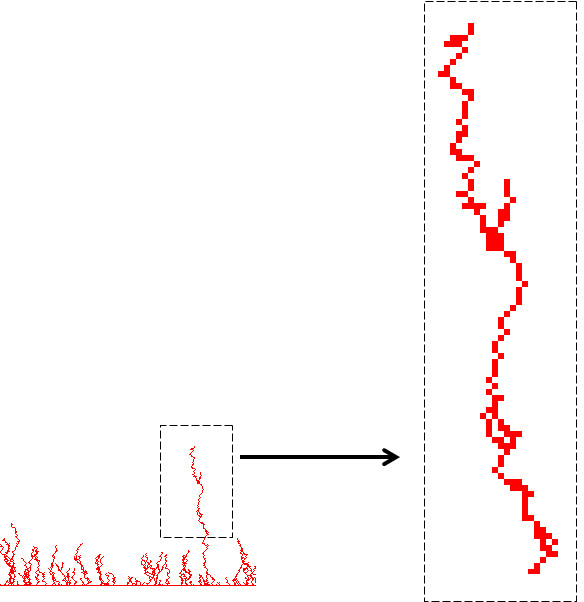
**Close-up view of a simulation outcome**. VEGF = 10000, sVEGFR-1 = 50000.

Experiments involving variable values of VEGF and sVEGFR-1 (R1, in Figure 3) produced biologically-consistent graphical outcomes and quantitative measurements. Figure 4 summarizes this first set of simulations. In each experimental setting 1000 simulations were implemented, each consisting of 100 cycles. The graphical outputs of these simulations recreate the branching and sprouting patterns observed in blood vessel network development. Similarly, the quantitative relationships observed between VEGF, sVEGR-1 and (mean) vessel network area are consistent with the expected responses: a. the higher the value of VEGF, with sVEGFR-1 constant, the larger the area covered by the resulting network (Figure 4A); b. the higher the value of sVEGFR-1, with VEGF constant, the smaller the observed network area (Figure 4B). Each panel also portrays snapshot examples of networks observed at the end of single simulations for various VEGF and sVEGFR-1 values. Figure 5 illustrates a close-up view of a simulation outcome.

**Figure 6:**
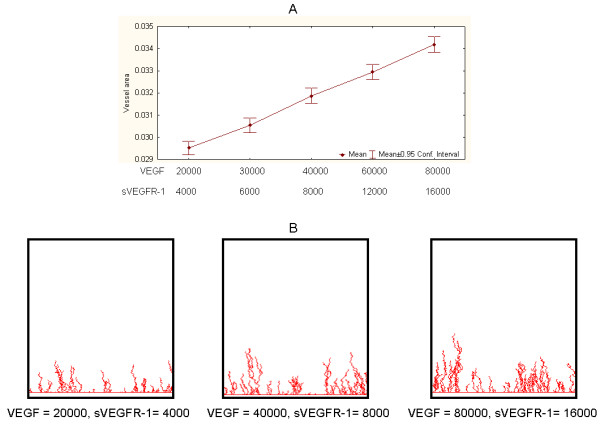
**Representative simulation results of biologically-plausible models**. Quantitative relationships and examples of output snapshots for different values of VEGF and sVEGFR-1, with sVEGFR-1/VEGF = 1/5. Model based on VEGF = 40000 and sVEGFR-1 = 8000 was chosen as a control model for implementing subsequent experiments.

Next, we aimed to implement models with more biologically-plausible VEGF and sVEGFR-1 values. This was done by focusing on experiments in which the sVEGFR-1/VEGF ratio was equal to 1/5, which is comparable to baseline values observed in *in vitro *control experiments. Figure 6 shows simulation results for several experimental settings, including examples of snapshots of graphical outcomes. As expected, vessel areas tend to proportionally and linearly depend on increases of both VEGF and sVEGFR-1 values, when sVEGFR-1/VEGF = 1/5. These experiments allowed us to propose a calibrated, biologically-plausible model that can be used as a control setting for subsequent analyses. We decided to focus on an experimental setting with VEGF = 40000 and sVEGFR-1 = 8000 as our control (or reference) model. This is a suitable selection because: a. it is based on a feasible concentration ratio value, b. its graphical outputs are sufficiently interpretable, and c. it leaves room (grid area) for possible significant enlargements or reductions in vessel network sizes in succeeding experiments.

### Model predictions

**Figure 7:**
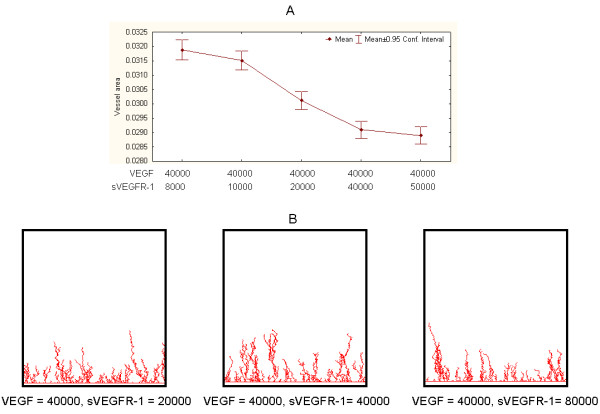
**Representative examples of effects of dynamically increasing sVEGFR-1 levels on vessel network development**. A. Vessel areas in response to different sVEGFR-1 values in comparison to control conditions (VEGF = 40000, sVEGFR-1 = 8000). B. Examples of simulation output snapshots.

We used the model to predict the effects of dynamically increasing sVEGFR-1 levels on vessel network development (R1, in Figure 3). Figure 7 illustrates representative quantitative and graphical results for different sVEGFR-1 values. The predicted outputs were compared to those obtained from control experiment (Figure 7A). As anticipated, the addition of sVEGFR-1 leads to reduction of vessel areas. However, significant departures from the mean area obtained under control conditions were detected when sVEGFR-1>1000 (experiment vs. control, t-tests, P < 1E-6). Smaller incremental changes were obtained when sVEGFR-1 ≥ 40000, suggesting a saturation of network reduction capacity.

**Figure 8:**
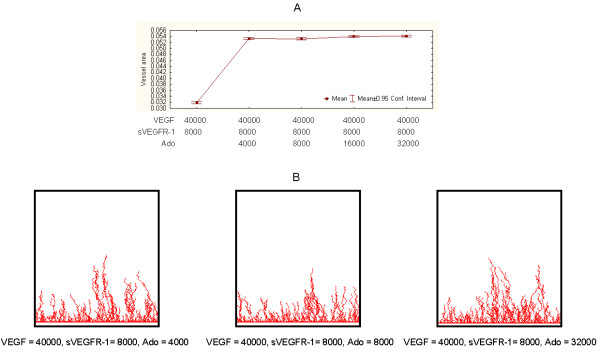
**Representative simulation results from Ado-mediated model**. A. Vessel areas in response to different Ado values compared to control conditions. B. Examples of simulation output snapshots.

Next we implemented independent sets of simulations to test the model involving exogenously added Ado (R2, in Figure 3). Our model does not incorporate endogenous Ado entities. This simplification is justified by the conditions of our *in vitro *experiments, which indicate that the level of endogenous Ado is much smaller than exogenously added Ado levels (Discussions). When inhibitors of Ado metabolism, such as EHNA (Methods), are added to the cell cultures, endogenous Ado accumulated in the medium is recycled towards AMP with the help of adenosine kinase [35]. Therefore, in the absence of cell breakdown or ischemia, the concentration of endogenous Ado is very low in cell cultures, including those composed of cardiac myocytes. As hypothesized and in comparison to controls, vessel area is increased by adding Ado (Figure 8). Such a difference was statistically significant (experiment vs. control, t-tests, P < 1E-6). Furthermore, and unexpectedly, such a tendency was observed even in conditions when added sVEGFR-1 was as twice as large as Ado (Figure 7A, second point on plot). Saturation of vessel area growth capacity appears to occur after Ado > 8000.

**Figure 9:**
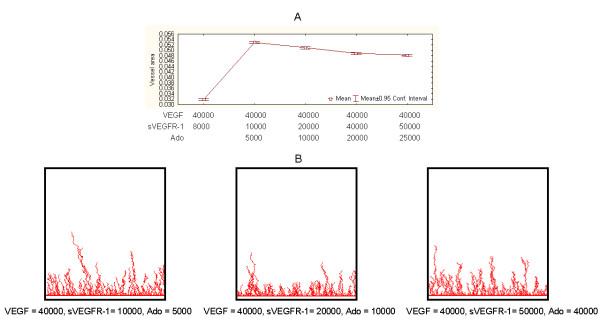
**Representative simulation results from models with different sVEGFR-1 and Ado values and sVEGFR-1/Ado = 2**. A. Vessel areas in response to increasing sVEGFR-1 and Ado values compared to control conditions. B. Examples of simulation output snapshots.

We further investigated the interplay between added sVEGFR-1 and Ado (combined) levels in biologically-viable conditions (R2, in Figure 3). We measured responses to different dynamic ranges of sVEGFR-1 and Ado under a sVEGFR-1/Ado ratio of 2 (Figure 9). This ratio conservatively mirrors the observation that in a single *in vitro *experiment sVEGFR-1 levels are larger, on average, than Ado levels (Discussions). Figure 9 corroborates the *in silico *findings reported above: adding Ado to the system results in increases of vessel network area in relation to controls, independently of added sVEGFR-1 levels for the proportions investigated. Such increases can be significant, with regard to control conditions, and even for small values of Ado relative to control VEGF levels. For Ado > 5000 and sVEGFR-1 > 8000, such differences are statistically detectable (control vs. experiments, t-tests, P < 1E-6). Strong differences, i.e., reductions, in terms of mean vessel areas between (pair-wise) experimental conditions were also detected (P < 1E-6), though less pronounced than the differences between these experiments and the controls.

### Adenosine effects on network reach

**Figure 10:**
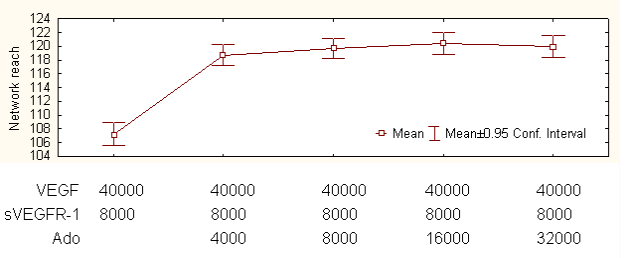
**The effect of Ado on network reach**. Network reach in response to different Ado values compared to control conditions.

Next we examined network "reach". This estimates how far a newly formed network can reach out from the initial vessel, i.e., the distance from the initial network to the farthest network tip. Thus, network reach also reflects the maximum branch length. We measured the effect of adding Ado on network reach for different amounts of Ado in comparison to the control setting. Results followed the same tendency observed in the case of network areas (Figure 10). Moreover, area and reach response are strongly, positively correlated in Ado-added experimental settings (Spearman correlation > 0.2, P < 0.05).

### Follow-up, exploratory *in vitro *experiments

**Figure 11:**
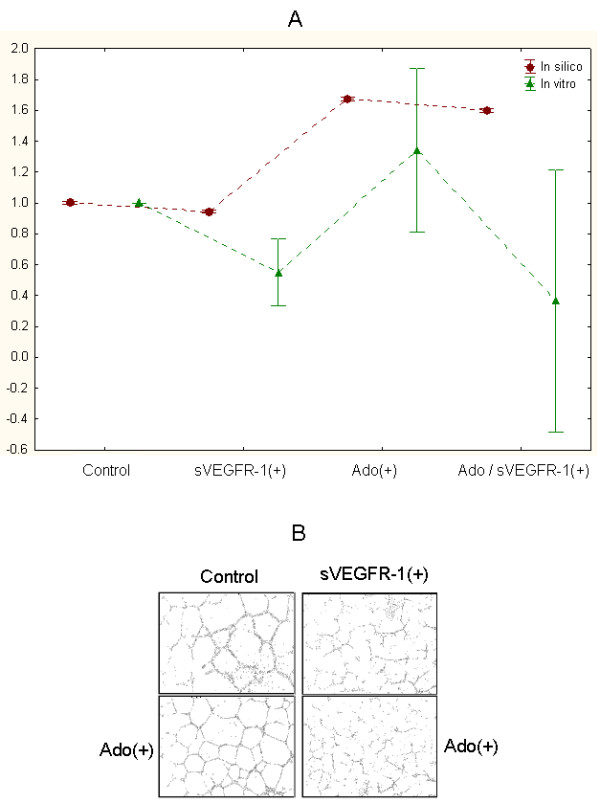
**Comparison of *in silico *and *in vitro *experiments**. A. Quantitative relationships between *in silico *and *in vitro *experiments across different experimental conditions on the basis of estimated vessel areas. Data are normalized to corresponding control mean vessel areas (points and bars: mean values of vessel areas ± 95% confidence interval). sVEGFR-1(+): added sVEGFR-1, Ado(+): added Ado, Ado(+)/sVEGFR-1(+): added combination of Ado and sVEGFR-1. B. Snapshot examples of the formation of (*in vitro*) microtubule networks in experiments using primary human coronary artery endothelial cells (HCAEC), and conditioned medium from macrophages stimulated for 24 hours by 100 ng/mL lipopolysaccharide. *In vitro *control condition: VEGF ~ 50 pg/mL, sVEGFR-1~ 10 pg/mL. Experimental conditions: Ado(+): 10 μmol/L, sVEGFR-1(+): 10 ng/mL.

As a starting point to motivate further development of the proposed computational model, we performed *in vitro *experiments using HCAECs in a Matrigel angiogenesis assay. The principle of this assay is to culture HCAEC in a conditioned medium from macrophages together with different treatments. The effect of the treatments on angiogenesis is measured by the surface of the culture dish occupied by microtubules formed by endothelial cells. We aimed to quantitatively compare the results obtained *in vitro *with this assay to our computational predictions on the basis of the observed vessel areas only (Figure 11).

Three *in vitro *experiments were completed in each of the following conditions: added sVEGFR-1, added Ado, and added combination of Ado and sVEGFR-1. In Figure 9, these experiments are referred to as sVEGFR-1(+), Ado(+) and Ado/sVEGFR-1(+) respectively. *In vitro *vessel areas were estimated in an automated fashion using densitometric analysis. *In vitro *control condition involved VEGF ~ 50 pg/mL and sVEGFR-1~ 10 pg/mL. The following values defined the experimental conditions: Ado(+) = 10 μmol/L and sVEGFR-1(+) = 10 ng/mL. The set of *in silico *experiments included here for comparison were implemented as follows: Control (VEGF = 40000 and sVEGFR-1 = 8000); added sVEGFR-1: VEGF = 40000, sVEGFR-1 = 20000; added Ado: VEGF = 40000, sVEGFR-1 = 8000, Ado = 4000; added combination of Ado and sVEGFR-1: VEGF = 40000, sVEGFR-1 = 20000, Ado = 10000. Similar correlation trends can be observed for different absolute values of sVEGFR-1 and Ado using control as a reference. Although we cannot state that there is a biologically-specific, one-to-one connection between these *in silico *parameters and their *in vitro *counterparts, we chose them for comparison purposes as they did not generate extreme simulation outcomes, i.e., neither maximum nor minimum network areas. Moreover, they are based on the biologically-feasible models presented above.

Overall, we found moderate quantitative, correlative associations between mean *in vitro *and *in silico *results (Spearman correlation = 0.4) across the different experimental conditions (Figure 11). As expected, in relation to control conditions, in both systems the incorporation of additional sVEGFR-1 induced a reduction of angiogenesis. Similarly, added Ado caused the formation of networks of larger areas.

Also in both models, adding a combination of Ado and sVEGFR-1 resulted in a reduction of mean vessel areas with respect to Ado(+) experiments. However, such a reduction was notoriously larger in the *in vitro *experiments. In contrast to the *in silico *model, we observed a mean vessel area below that obtained in control conditions. Although the small number of *in vitro *experiments represent a limitation to a more accurate quantitative comparison, such an *in vitro *vs. *in silico *difference was statistically detectable at P = 1E-3. This discrepancy may suggest new research directions or limitations of our computational model (Discussion). Figure 11B depicts examples of graphical snapshots of different *in vitro *experimental conditions.

## Discussion

Previous research has shown the potential pro-angiogenic role of Ado and its clinical relevance to heart disease [23-25]. Here we reported computational, rule-based dynamic simulations of angiogenesis in which Ado, as well as known ECs growth promoting and inhibiting factors, were modelled to investigate biologically-meaningful responses. In addition, our findings point to possible new research directions in this area. In particular, we tested *in silico *the hypothesis that Ado may step-up angiogenesis through supporting VEGF activity and counteracting sVEGFR-1. Although related, our method is not in the strict sense based on a cellular automata model. More specifically, it falls into the category of agent- or individual-based systems, which can be defined as a generalization of the cellular automata approach.

Angiogenesis is under the control of a plethora of pro- and anti-angiogenic factors [1] and is therefore a very complex phenomenon subjected to intense regulation. It is sometimes difficult from biological assays alone to determine the net, global effects of candidate molecules on angiogenesis. Computational approaches, such as the one described here, can aid biologists in designing experiments and draw conclusions. Alternatively, some molecules share both pro- and anti-angiogenic properties and computational models may help to determine the dominant effect. Finally, this effect is highly dependent on the concentration of the molecules and its vicinity, i.e., the amounts of pro- and anti-angiogenic molecules supposed to interact together to dictate a pro- or anti-angiogenic signal. Again, computational models can help researchers to estimate the concentration of molecules to be used in *in vitro *tests, together with other factors that should be incorporated into the tests. We believe that our present data on adenosine and sVEGFR-1 illustrate these limitations inherent to *in vitro *tests.

### Most relevant insights and advances

Our models are based on the dynamic interaction of cells and molecular entities encoded as autonomous, discrete system components or agents. These locally-occurring interactions are specified by a set of logical (if-then) rules that give rise to global, emergent behaviour throughout thousands of time cycles and simulations. These and alternative approaches are suitable to tackle the complexity and broad knowledge scope of angiogenesis research, in general, and of a cardiovascular disease framework, in particular. Our model was capable to generate visual quantitative patterns and predictions that are entirely derived from the system's self-organizing properties, as well as from adaptations to several stimuli. These features also make our approach appealing to the non-specialist user of computational tools or to those less proficient in mathematical formalisms.

The main objective of our study was to assess the potential of the proposed approach as a simulation-based exploratory tool. Thus, the implementation of a rule-based methodology can also be justified by its openness and flexibility: it does not require the precise approximation of mathematical parameters, such as concentration rates, which are needed in traditional reaction models. At this stage, this is particularly relevant due to the relative lack of quantitative information to allow us to implement more detailed models of the molecules investigated.

For constant VEGF values, added Ado can induce stronger (absolute) changes in network area than those prompted by sVEGFR-1's increases over comparable variation scales (Figures 7 and 8). In each scenario the induced absolute changes (from minimum to maximum areas) are notable: ~10% vs. ~68% for sVEGFR-1 and Ado variation scenarios respectively. This offers quantitative evidence to further characterize the angiogenesis hypothesis underlying our model. That is, VEGF's pro-angiogenic effect on a systems level is enhanced by its local interplay with Ado, regardless of the presence of sVEGFR-1. Conversely, the global inhibitory power of sVEGFR-1 is conditioned to the absence of Ado in local neighbourhoods.

We also presented a comparative assessment of the resulting models in relation to *in vitro *experiments using human ECs. Although we cannot consider this as an experimental validation of our approach, it offers preliminary evidence about its potential to recapitulate several angiogenesis-like principles and patterns. Researchers can also interrogate our model using different types of quantitative "what-if" queries based on specific molecular parameters controlling ECs network growth and sprouting. Perhaps most important, the model has improved our knowledge of Ado-mediated angiogenesis in different biologically-relevant conditions.

The partial disagreement observed in the *in silico *vs. *in vitro *comparisons (the effect of the combined action of added Ado and VEGRF-1), also provides us with significant insights. The reported discrepancy may be explained by the relative small number of *in vitro *experiments conducted. Also we cannot exclude the possibility of the influence of other angiogenic factors present in cell culture medium that were not considered in the *in silico *approach and that could interact with the added molecules. In addition, it has to be considered that the three *in vitro *replicates were independent experiments with different cell batches and conditioned medium from different cultures of primary human cells (therefore containing different mixtures of angiogenic factors and cells with different genetic background). These limitations support the vessel network area variability detected in our preliminary *in vitro *results. Nevertheless, these results may also highlight knowledge gaps in our computational model and prior hypotheses. This also means that our results are already pointing to a specific aspect of Ado-mediated angiogenesis that requires further investigations. This may imply the need for a re-formulation of our core hypothesis about the role of Ado in the conditions studied here. For example, this could require us to consider the incorporation of additional molecular entities into our model, such as those whose interaction are required in control conditions or in the specific context of added Ado/sVEGFR-1.

Thus we can postulate that, although computational experiments may not account for all observed *in vitro *behaviour, the models investigated here and future extensions can assist us in testing and generating biologically-relevant hypotheses. This can additionally help researchers to make informed decisions on future experimental research. The latter is based on the idea that these models can explore experimental settings that can be more expensive or challenging to deploy in a systematic manner in the "wet lab".

### Limitations and future research

A possible limitation of algorithmic, discrete models is the difficulty in defining specific model parameters, such as concentration values. Our models underwent a verification phase that resulted in responses that were consistent with expected control responses. In this case the selection of parameters was guided by experimentally-meaningful relationships, such as concentration value proportions between molecular entities in control conditions. The challenge of numerical specification of parameters in algorithmic, discrete models is not as great as that posed in the design of traditional, mathematical models (i.e., differential equations).

One of the possible next steps in our research is to achieve a tighter linkage between *in silico *and *in vitro *experiments, which can cover alternative qualitative and quantitative predictive features. Although in this study we focused on a phenotypic characterization based on vessel network areas, it is important to design models that can provide a better approximation to the graphical details (detailed vascular patterning) of the specific *in vitro *conditions. Solutions, however, will inevitably depend on the biological questions asked by the researchers, as well as on the biological cultures and assays available. We would also expect to implement additional *in vitro *experiments with larger sample sizes or alternative protocols.

As a first step in a proof-of-concept investigation, we focused on global quantitative relationships between *in silico *and *in vitro *models. In particular, the total area of the resulting networks represented the quantitative criterion to test our models and establish correlations between them. This is a critical modelling factor that needs to be addressed before refining models to assess other quantitative or qualitative relationships. Qualitative aspects, such as a more detailed reproduction of visual patterns, will also require additional *in vitro *work. In this study, for example, we applied an endothelial microtube formation assay as a first *in vitro *evaluation step. In contrast to our *in silico *models, in this assay an artery segment was not used as the initial vascular network. A more accurate correlation of qualitative features, e.g., shape of the networks, could involve the implementation of an assay in which an artery segment is used as the seed vascular network. Building on the outcomes of this investigation, in the future we aim to implement new *in vitro *experiments to assess additional quantitative and qualitative correlative indicators.

Our model synthesizes experimentally-feasible mechanisms at the cellular level. Thus, future applications may involve more specific behaviours such as protein secretion, detachment of ECs from existing vessels and EC proliferation in response to different types of chemoattractants. The simulations reported here excluded the specification of endogenous Ado because in our *in vitro *conditions its levels tend to be negligible in comparison to the levels of added Ado. Although future research may soften this assumption by allowing the incorporation of endogenous Ado, we do not expect this simplification to have a significant influence on the overall findings of this research. Another simplification was that the (*in silico*) time scale was assumed to be sufficiently shorter to keep entity levels constant. As part of future research, it would be important to implement simulations whose time scales can more accurately correspond to *in vitro *scales. Moreover, our model can be adapted to represent time-dependent degradation of molecular entities, such as Ado, whose half-life is known to be short. Future research will also attempt to model more realistic spatial and entity geometrical aspects.

The rationale behind the 2-D grid is that it approximates the spatial representation of a typical *in vitro *angiogenesis assay, i.e. 2-D models based on Matrigel. Variations of the topological assumptions, e.g. moving to a 3-D model, should not be expected to cause significant differences at the systems response level. Nevertheless, we would agree that this will naturally increase the computational complexity of our model implementations. In addition, this would require new, typically more expensive ways, to validate our results *in vitro*. Although at this stage we are focusing on a 2-D grid model due to its biological relevance and existing technical constraints, as part of future work we will definitely consider extensions of our model to accommodate variations in spatial constraints.

The intrinsic assumptions of our model logic were those at the core of our biological hypotheses, which in turn were motivated by prior knowledge. Hence, at this stage it would be difficult to point to a specific logical aspect that could accurately explain the observed discrepancy. Possible explanations will be obtained as new model configurations (hypotheses) will be tested using our simulation system. In the light of the outcomes of this pilot study, we are considering the possibility of refining our models by incorporating additional entities and logical relations not encoded here. However, this effort is still in a planning stage and will require significant biological literature examination and model requirements analysis. A crucial requirement for selecting a new hypothesis for computational modelling will be its biological viability and potential relevance. Furthermore, this will also depend on the identification of feasible strategies and resources for the *in vitro *validation of the resulting *in silico *predictions.

## Conclusions

Despite the need for achieving deeper levels of biological detail and for additional wet-laboratory work to further assess our findings, our approach offers a new knowledge discovery-support tool. Such a tool can improve our understanding of a clinically-relevant problem at a systems- and multi-cell level. This and related algorithmic modelling approaches will be vital to cost-effectively engineer novel patient-intervention and drug discovery strategies in a systems biomedicine era. This research exemplifies the application of discrete computational models to explore hypotheses and inform biological research, and provides a foundation from which to enable deeper understandings of angiogenesis.

## Competing interests

The authors declare that they have no competing interests.

## Authors' contributions

FA: Conceived the investigation, implemented computational experiments and wrote the manuscript assisted by the other authors. FL and MRT: Performed *in vitro *experiments. YD and DW provided biological interpretations. All authors read and approved the final manuscript

## Supplementary Material

Additional file 1 **Model responses to different numbers of cycles/simulation**. Data plot. Click here for file
