# Supplementary material for: Proof-of-principle investigation of an algorithmic model of adenosine-mediated angiogenesis
Source: Theor Biol Med Model. 2011 Apr 8;8:7. doi: 10.1186/1742-4682-8-7 (PMC3090359; doi:10.1186/1742-4682-8-7)
Supplement: Additional file 1 — Model responses to different numbers of cycles/simulation. Data plot. [file 1742-4682-8-7-S1.PDF]

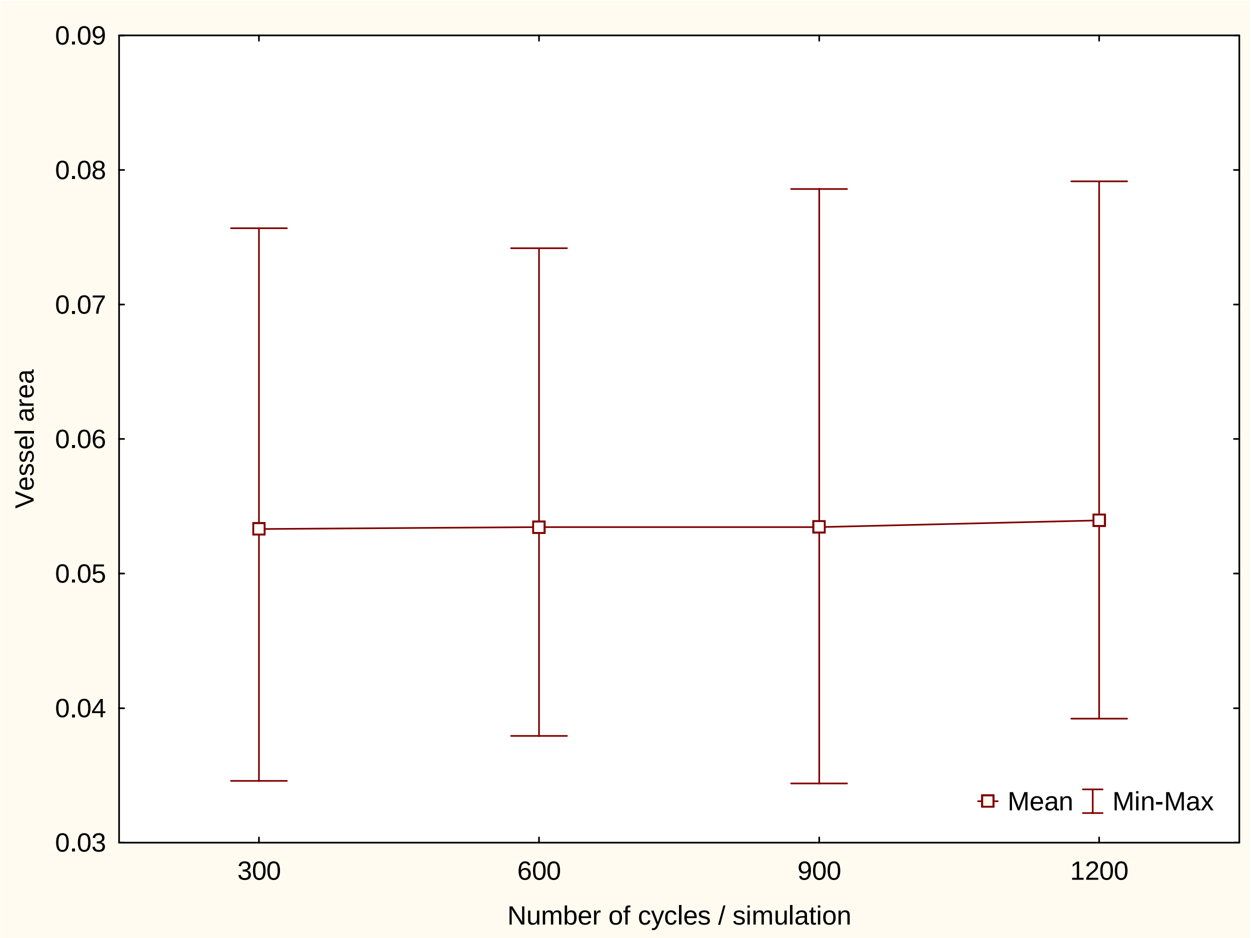

Figure S1. Model responses to different numbers of cycles / simulation. Model parameters: VEGF = 40000, VEGFR-1= 8000, Ado = 4000, 1000 simulations.
